# Beyond the metropolises: the decentralization of chikungunya to non-metropolitan areas across Brazil

**DOI:** 10.1590/0102-311XEN129025

**Published:** 2026-07-06

**Authors:** Iasmim Ferreira de Almeida, Cláudia Torres Codeço, Raquel Martins Lana

**Affiliations:** 1 Escola Nacional de Saúde Pública Sergio Arouca, Fundação Oswaldo Cruz, Rio de Janeiro, Brasil.; 2 Programa de Computação Científica, Fundação Oswaldo Cruz, Rio de Janeiro, Brasil.; 3 Barcelona Supercomputing Center, Barcelona, España.

**Keywords:** Chikungunya Virus, Arbovirus Infections, Incidence, Vírus Chikungunya, Infecções por Arbovírus, Incidência, Virus Chikungunya, Infecciones por Arbovirus, Incidencia

## Abstract

Chikungunya virus (CHIKV) was first detected in Brazil in 2014, becoming a significant public health concern. Metropolitan areas were initially the port of entry, but the disease spread to less populated municipalities, challenging the focus of monitoring and control in large cities. This study presents a descriptive analysis of the spread of CHIKV in Brazil up to 2022, examining regional differences. Data on chikungunya cases in municipalities were obtained from the Brazilian Information System for Notifiable Diseases (SINAN) from 2014-2022. Population size data were obtained from the Brazilian Institute of Geography and Statistics (IBGE). The chikungunya introduction dates in the states were organized into timelines and maps. A decentralization index was proposed to estimate the ratio between the incidence in non-metropolitan and metropolitan municipalities. Our analysis revealed that the spread of chikungunya from metropolitan to non-metropolitan areas varied among states. As expected, most states initially showed higher metropolitan incidence rates, with a few exceptions, such as Amapá, located at the national border with French Guiana. The Northeast remained the epicenter during the study period, but significant regional differences in disease patterns were observed across Brazil. Several factors, including environmental suitability and demographic changes such as internal migration, may have facilitated the differential spread of chikungunya to less densely populated regions. In conclusion, the index proved useful for monitoring the dynamics of disease spread, highlighting areas that require specific surveillance and control measures extending beyond large urban centers.

## Resumo

O vírus Chikungunya (CHIKV) foi detectado pela primeira vez no Brasil em 2014, tornando-se uma preocupação de saúde pública relevante. Inicialmente, as regiões metropolitanas serviram como porta de entrada, mas a doença disseminou-se para municípios menos populosos, desafiando as estratégias de monitoramento e controle focadas nas grandes cidades. Este estudo apresenta uma análise descritiva da disseminação do CHIKV no Brasil até 2022, examinando as diferenças regionais. Os dados referentes aos casos de chikungunya nos municípios foram extraídos do Sistema de Informação de Agravos de Notificação (SINAN) para o período de 2014 a 2022. Os dados de tamanho populacional foram obtidos do Instituto Brasileiro de Geografia e Estatística (IBGE). As datas de introdução da chikungunya nos estados foram organizadas em linhas do tempo e mapas. Propôs-se um índice de descentralização para calcular a razão entre a incidência nos municípios não metropolitanos e metropolitanos. A análise revelou que a disseminação da chikungunya das regiões metropolitanas para as não metropolitanas variou entre os estados. Como esperado, a maioria dos estados apresentou inicialmente taxas de incidência metropolitana mais elevadas, com algumas exceções, como o Amapá, situado na fronteira com a Guiana Francesa. O Nordeste manteve-se como epicentro durante o período do estudo, mas diferenças regionais significativas nos padrões da doença foram observadas em todo o país. Diversos fatores, incluindo a suscetibilidade ambiental e mudanças demográficas como a migração interna, podem ter facilitado a disseminação diferencial da chikungunya para regiões menos densamente povoadas. Em conclusão, o índice demonstrou ser útil para monitorar a dinâmica de disseminação da doença, evidenciando áreas que demandam medidas específicas de vigilância e controle para além dos grandes centros urbanos.

## Resumen

El virus Chikungunya (CHIKV) se detectó por primera vez en Brasil en 2014, convirtiéndose en un importante problema de salud pública. Las regiones metropolitanas fueron inicialmente el punto de entrada, pero la enfermedad se propagó a municipios menos poblados, lo que supuso un reto para la vigilancia y el control en las grandes ciudades. Este estudio presenta un análisis descriptivo de la propagación del CHIKV en Brasil hasta 2022, examinando las diferencias regionales. Los datos sobre los casos de chikungunya en los municipios se obtuvieron del Sistema de Información de Enfermedades de Notificación Obligatoria (SINAN) entre 2014 y 2022. Los datos sobre el tamaño de la población se obtuvieron del Instituto Brasileño de Geografía y Estadística (IBGE). Las fechas de introducción del chikungunya en los estados se organizaron en cronologías y mapas. Se propuso un índice de descentralización para calcular la relación entre la incidencia en los municipios metropolitanos y no metropolitanos. Nuestro análisis reveló que la propagación del chikungunya desde las regiones metropolitanas a las no metropolitanas variaba entre los estados. Como era de esperar, la mayoría de los estados mostraron inicialmente tasas de incidencia más altas en las zonas metropolitanas, con algunas excepciones, como Amapá, situado en la frontera nacional con la Guayana Francesa. El noreste siguió siendo el epicentro durante el período de estudio, pero se observaron diferencias regionales significativas en los patrones de la enfermedad en todo Brasil. Varios factores, entre ellos la idoneidad del entorno y los cambios demográficos, como la migración interna, pueden haber facilitado la propagación diferencial del chikungunya a regiones menos densamente pobladas. En conclusión, el índice resultó útil para supervisar la dinámica de la propagación de la enfermedad, poniendo de relieve las zonas que requieren medidas específicas de vigilancia y control más allá de los grandes centros urbanos.

## Introduction

Based on the literature and data from Brazilian surveillance systems, the Chikungunya virus (CHIKV) was first detected in Brazil in 2014. Only genetic analyses conducted in 2015 confirmed the presence of two distinct lineages: the Asian genotype in Oiapoque, Amapá (Northern Region), and the East/Central/South African (ECSA) genotype in Feira de Santana, Bahia (Northeast Region) ^1^. Since then, the ECSA lineage has become predominant in all Brazilian regions ^2^ ^,^ ^3^. Some studies suggest that CHIKV may have been introduced earlier, primarily through imported cases. For instance, researchers propose that the virus may have entered the country up to a year before its official detection ^4^ ^,^ ^5^.

These initial disagreements probably stem from multiple factors, including the similarity of chikungunya symptoms to other arboviruses, such as dengue, which was already widely known, and the initial lack of specific diagnostic tests for CHIKV in Brazil ^4^ ^,^ ^5^. In 2016, two years after the notification of the first case, the highest number in the historical series was recorded, with 277,500 reported cases. However, in 2024, a substantial increase of 68.2% was observed compared to 2023, rising from 158,060 to 265,882 cases. This makes 2024 the year with the second-highest number of reported cases since the beginning of the series, highlighting a concerning trend ^6^. CHIKV has been circulating in the Americas since 2013, with experts warning of its potential emergence in Brazil ever since ^7^ ^,^ ^8^. Their concerns included the high infestation levels of *Aedes aegypti* and *Aedes albopictus* (also vectors for dengue and Zika), dengue virus endemism, the detection of mutations that enhance transmission by *Ae. albopictus*, and the universal susceptibility of the Brazilian population to this novel virus ^9^. Moreover, global mobility and climate change have heightened these risks, underscoring the need for vigilance and the prevention of outbreaks ^10^. *Aedes*-transmitted arboviruses are primarily urban, occurring in densely populated regions ^11^ ^,^ ^12^ ^,^ ^13^ where habitats conducive to mosquito productivity are abundant ^3^ ^,^ ^14^.

In Brazil, urban regions can vary from large megalopolises, such as São Paulo and Rio de Janeiro, to small towns with a few thousand individuals. The former are more susceptible to disease importation due to the large flow of individuals and materials that potentially carry infected mosquitoes in aquatic or adult stages, which is exacerbated by poor sanitation and high population density ^15^.

Studies on dengue spatial dispersion reveal a pattern that starts in metropolitan areas and spreads to towns in peripheral municipalities located in non-metropolitan areas, characterized by smaller populations and more limited influence ^14^ ^,^ ^16^. We hypothesize that the same pattern should apply to chikungunya. The process of spatial decentralization in the occurrence of diseases has been described in various contexts, through the mapping of municipal-level HIV incidence rates ^17^ or the detection of spatiotemporal clusters of yellow fever ^18^.

In this context, this study aimed to characterize the dissemination pathways of chikungunya across Brazil from its emergence until 2022. We propose easily computable metrics, such as the decentralization index, capable of continuously capturing the temporal dynamics of decentralization and strengthening spatial monitoring within the scope of health surveillance.

## Methods

### Study region

Brazil is a country of continental dimensions, encompassing several distinct climate types, including equatorial, tropical, semiarid, highland, subtropical, and humid coastal, due to its latitudinal range, which varies approximately between 5°N and 33°S. It is organized into 27 Federative Units (UF, acronym in Portuguese), comprising 26 states and the Federal District (Brazil’s capital, Brasília), distributed across five regions: North, Northeast, Central-West, Southeast, and South ^17^. Each UF comprises municipalities, with population sizes ranging from 12.4 million inhabitants in São Paulo to 833 in Serra da Saudade (Minas Gerais) ^19^.

The Brazilian Institute of Geography and Statistics (IBGE, acronym in Portuguese) classifies municipalities according to a hierarchy of influences in several categories, the REGIC (Region of Influence of Cities) ^19^, based on the flow of people, goods, and services. In this study, a new classification of municipalities was developed by grouping the REGIC categories into two groups: metropolitan and non-metropolitan. This approach aimed to simplify the analysis of territorial inequalities and to better understand how the level of urban centrality may influence the patterns of arbovirus dissemination in the country.

The metropolitan municipalities encompassed metropolises with national influence (São Paulo and Rio de Janeiro in the Southeast, and the Federal District in the Center-West) and metropolises with regional influence, which include some of the state capitals (Belém [Pará State] and Manaus [Amazoans State] in the North Region; Fortaleza [Ceará State], Recife [Pernambuco State], and Salvador [Bahia State] in the Northeast Region; Goiânia [Goiás State] in the Center-West, Vitória [Espírito Santo State] and Campinas [São Paulo State] in the Southeast; and Porto Alegre [Rio Grande do Sul State], Florianópolis [Santa Catarina State], and Curitiba [Paraná State] in the South Region). The metropolitan group also included municipalities classified in the second and third hierarchical levels of REGIC, specifically regional capitals and sub-regional centers A, with populations exceeding 150,000 inhabitants. Although these municipalities have a significant concentration of administrative functions, they exhibit a more circumscribed sphere of influence compared to the core metropolises ^20^. Thus, a total of 443 municipalities were included in the group of metropolises, as defined by the study criteria. Non-metropolitan municipalities include the other municipalities, presenting a large variation in population size, ranging from a minimum of 833 inhabitants to a maximum of 400,000 inhabitants ^19^ ^,^ ^20^, totaling 5,127 municipalities.

### Data

Epidemiological data were obtained from the Brazilian Information System for Notifiable Diseases (SINAN, acronym in Portuguese) ^21^, downloaded from InfoDengue for the years 2014 and 2022 ^22^ and from 2015 to 2021, extracted using the R package (http://www.r-project.org) “microdatasus” ^23^, accessing the SINAN database directly via tile transfer protocol (FTP).

The dataset is available in public repositories, and, according to Brazilian law, its use does not require approval from an ethics committee.

Reported chikungunya cases, by definition, are those in which patients presented with sudden-onset fever and severe arthralgia or arthritis of acute onset, unexplained by other conditions, resided in or traveled to endemic or epidemic areas up to 14 days before the onset of symptoms, or had an epidemiological link with a confirmed imported case ^21^. Exclusion criteria encompassed cases marked as discarded (cases initially identified as chikungunya and later discarded for any reason) and cases residing in another country. The remaining cases, referred to as probable cases, included confirmed cases, as determined by both laboratory and clinical-epidemiological criteria, as well as cases under investigation.

Population projections for the study period, estimated by the IBGE, were obtained through Brazilian Health Informatics Department (DATASUS, acronym in Portuguese) for each year of the analysis period ^24^.

### Data analysis

Time series data on probable chikungunya cases were organized by notification date to visualize their spread throughout Brazil at the UF level. A timeline indicates the initial month of the first autochthonous case notification by UF. To describe the magnitude of chikungunya activity in the country over the period, the number of new municipalities reporting cases, and accumulated cases by year and region were computed and presented in tables and maps.

### Decentralization index (*ψ*)

The decentralization index (*Ψ(y,s)*) is a new indicator proposed in this study to describe the hypothesized spread of chikungunya between metropolitan (*M*) and non-metropolitan (*N*) areas within each UF (*s*), by year (*y*). This metric estimates the ratio between the total incidence in non-metropolitan municipalities (*IN(y,s)*) and the total incidence in metropolitan municipalities (*IM(y,s)*), for each state and year, with higher values reflecting a greater degree of decentralization in disease transmission.

The formula for the decentralization index (*Ψ*):

(1) $$\Psi =\frac{ΙΝ\left(\text{y,s}\right)}{ΙΜ\left(\text{y,s}\right)}$$

(2) $$ΙΝ\left(\text{y,s}\right)=\frac{\sum m\notin N_{s}C\left(\text{m,y}\right)}{\sum m\notin N_{s}P\left(\text{m,y}\right)}$$

(3) $$ΙΜ\left(\text{y,s}\right)=\frac{\sum m\in M_{s}C\left(\text{m,y}\right)}{\sum m\in M_{s}P\left(\text{m,y}\right)}$$

In which *C(m, y)* is the total number of cases in municipality *m* in year *y*, and *P(m, y)* is the population in *m* in year *y*; Ns is the set of non-metropolitan municipalities in each federative unit (*s*); and *Ms* is the set of metropolitan municipalities in each UF (*s*). For each UF (*s*), the hypothesis was that the initial incidence of chikungunya cases was higher in metropolitan area (*Ψ < 1*) and that, over time, cases moved to non-metropolitan area (*Ψ > 1*). The index ranges from 0 to *∞*, where values close to 0 reflect an extreme concentration in metropolitan area, while values above 1 indicate a significant redistribution toward non-metropolitan area. A value equal to 1 represents a homogeneous distribution of incidence between metropolitan and non-metropolitan area, indicating balance in the spatial distribution of cases. All analyses and visualizations were performed using R software version 4.3.1 (http://www.r-project.org).

## Results

### Introduction and initial spread of chikungunya in Brazil

**Figure 1:**
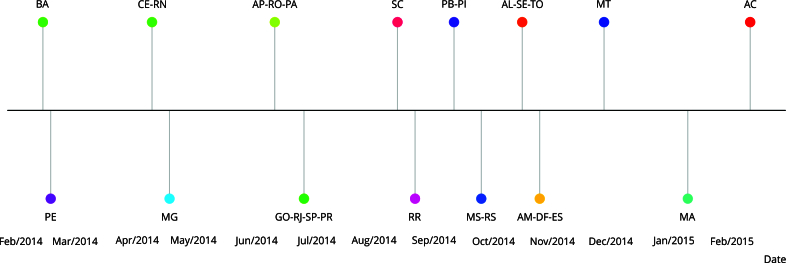
Timeline of the first autochthonous chikungunya case in each Brazilian Federative Unit by month, from January 2014 to February 2015.

Note: regions and their respective states: North − AC: Acre, AM: Amazonas, AP: Amapá, PA: Pará, RO: Rondônia, RR: Roraima, and TO: Tocantins; Northeast − AL: Alagoas, BA: Bahia, CE: Ceará, MA: Maranhão, PE: Pernambuco, PB: Paraíba, PI: Piauí, RN: Rio Grande do Norte, and SE: Sergipe; Central-West − DF: Federal District, GO: Goiás, MS: Mato Grosso do Sul, and MT: Mato Grosso; Southeast − ES: Espírito Santo, MG: Minas Gerais, RJ: Rio de Janeiro, and SP: São Paulo; South − PR: Paraná, RS: Rio Grande do Sul, and SC: Santa Catarina. Y-axis scales vary across panels to enhance visualization of state-specific patterns.

According to the SINAN database, the first reports of autochthonous chikungunya cases were recorded in February 2014, simultaneously in the states of Bahia and Pernambuco, in the Northeast Region (Figure 1). 

Subsequently, there were reports in Ceará and Rio Grande do Norte (Northeast) and Minas Gerais (Southeast Region), which borders Bahia. This is a predominantly semiarid region, characterized by high temperatures ^25^ ^,^ ^26^ and low humidity throughout the year, except during the rainy season from March to July, when weather conditions favor the development of *Aedes* mosquitoes ^27^. These states have only two metropolises, the capitals Fortaleza (Ceará) and Belo Horizonte (Minas Gerais), and the capital of Rio Grande do Norte (Natal); the other municipalities that make up the states are not considered to have great influence according to the REGIC classification ^20^. Most of the population in the region suffers from structural issues with their water supply, and domestic water storage containers have been identified as important breeding grounds for mosquitoes ^28^.

In June 2014, four months after the initial cases, seven other states throughout the country (Amapá, Rondônia, and Pará in the North; Goiás in the Central-West; Rio de Janeiro and São Paulo in the Southeast; and Paraná in the South) reported autochthonous cases. This arrival coincided with an atypical period for arboviruses, as June is typically an off-season for dengue due to lower temperatures and reduced mosquito activity ^29^. By the end of 2014, 25 of the 27 states had reported chikungunya cases, totaling 302 municipalities with 1,769 probable cases and an average incidence of 2.14 cases per 100,000 inhabitants (Table 1). In this initial year, 71.7% of the cases were registered in the North and Northeast regions of the country (1,269 cases).

**Table 1:** Probable cases, incidence rate (per 100,000 inhabitants), and total municipalities with at least one probable autochthonous chikungunya case per year by region from 2014 to 2022.

Regions/Variables	2014	2015	2016	2017	2018	2019	2020	2021	2022	Cumulative total
North										
Municipalities	20	55	144	75	21	11	10	15	15	366
Cases	429	1,800	8,339	16,788	8,760	4,022	673	1,340	4,960	47,111
Annual incidence	1.73	6.42	57.06	90.21	18.48	12.76	3.84	14.36	49.96	28.31
Northeast										
Municipalities	113	419	719	127	43	64	43	45	77	1,650
Cases	840	29,483	178,595	143,854	11,526	34,031	59,273	67,898	148,679	674,179
Annual incidence	0.67	46.13	285.48	109.67	13.74	38.27	78.41	89.32	319.48	109.02
Southeast										
Municipalities	120	134	328	149	113	168	64	53	62	1,191
Cases	343	1,448	8,231	23,083	53,511	92,589	12,461	25,701	14,682	232,049
Annual incidence	0.28	0.76	5.40	21.45	20.97	42.21	15.25	16.05	22.32	16.08
South										
Municipalities	25	80	213	39	25	36	38	24	55	535
Cases	45	328	2,932	299	231	416	557	628	709	6,145
Annual incidence	0.07	0.68	9.51	0.82	0.70	1.36	2.02	4.90	3.64	2.63
Central-West										
Municipalities	24	68	88	61	42	20	17	21	31	372
Cases	112	921	2,403	3,999	14,058	1,002	730	1,379	6,020	30,624
Annual incidence	0.34	5.60	16.93	8.58	28.35	6.36	4.85	14.35	28.41	12.64
Brazil										
Municipalities	302	756	1,492	451	244	299	172	158	240	4,114
Cases	1,769	33,980	200,500	188,023	88,086	132,060	73,694	96,946	175,050	990,108
Annual incidence	2.14	28.7	123	127	62.8	85.6	52.6	68.6	113	-

Note: metropolitan municipalities - North = 50, Northeast = 117, Southeast = 128, South = 95, Central-West = 54, Brazil = 444; non-metropolitan municipalities - North = 400, Northeast = 1,677, Southeast = 1,540, South = 1,096, Central-West = 413, Brazil = 5,126.

In February 2015, the remaining states, Maranhão and Acre, detected their first cases. Similarly, Acre was also one of the last states to detect the presence of dengue in Rio Branco, the state capital. This delay was attributed to the lower centrality of Acre compared to other states ^30^. In contrast, Maranhão has historically had a low dengue notification record, which may indicate either low transmission or deficiencies in epidemiological surveillance ^30^ ^,^ ^31^. In 2015, the cumulative incidence of chikungunya in the country increased 13-fold compared to the previous year, reaching 28.7 cases per 100,000 inhabitants (Table 1). The Northeast region remained the epicenter of the disease, with 419 new municipalities reporting a total of 29,483 (86.8%) cases, a 69-fold increase compared to the previous year (annual cumulative incidence of 46.13 per 100,000 population) (Supplementary Material; https://cadernos.ensp.fiocruz.br/static//arquivo/suppl-e00129025_7821.pdf). The South and Southeast regions recorded the lowest incidences in 2015, 0.68 and 0.76 per 100,000 population, respectively.

The year 2016 witnessed an even more significant spread nationally, with an approximately six-fold increase in cases (200,500) and a four-fold increase in incidence (123 per 100,000 population) compared to the previous year. The Northeast remained the epicenter, accounting for 89% of all reported cases and reaching the peak of the historical series for the region with 178,595 cases. All regions observed an increase in cases, and approximately 1,492 municipalities reported a probable case for the first time. In 2017, the number of cases remained high (188,023 cases), with an incidence of 127 cases per 100,000 inhabitants, showing an increasing trend in the North (16,788), Central-West (3,999), and Southeast (23,083), and a reduction in the Northeast (143,854) and South (299) (Supplementary Material, Figure S1; https://cadernos.ensp.fiocruz.br/static//arquivo/suppl-e00129025_7821.pdf).

In 2018, there was a downward trend in cases nationwide, with 88,086 cases (62.8 cases per 100,000 inhabitants), particularly in the Northeast, which accumulated around 10,000 cases in that year. However, while the North and Northeast saw a decrease in cases and incidence, regions such as the Southeast experienced a significant increase, accounting for 61% of the country’s cases (53,511). A similar pattern was registered in 2019.

The years 2020 to 2022 were atypical due to the COVID-19 pandemic. In 2020, there was a notable reduction in chikungunya cases (73,694) in Brazil, possibly due to social distancing and hygiene practices adopted, which also affected the dynamics of other infections ^32^ ^,^ ^33^. Additionally, the overload of health services may have prioritized COVID-19 notifications over those for arbovirus infections ^33^ ^,^ ^34^. In 2021, despite the ongoing pandemic, there was a slight increase in cases in all regions. In 2022, there was a significant resurgence of cases in all regions except the Southeast. By this year, chikungunya had been detected in all UF, with 73.9% (4,114) of municipalities reporting at least one probable case, and more than 990,000 cases accumulated during the study period (2014-2022).

### Decentralization of chikungunya incidence

**Figure 2:**
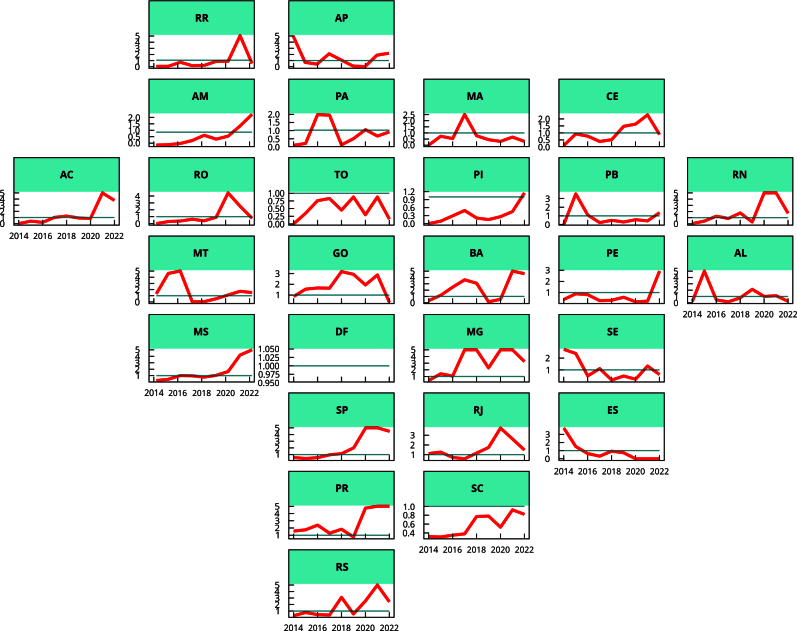
Chikungunya decentralization index (*Ψ*) by year and Brazilian Federative Unit.

Note: regions and their respective states: North − AC: Acre, AM: Amazonas, AP: Amapá, PA: Pará, RO: Rondônia, RR: Roraima, and TO: Tocantins; Northeast − AL: Alagoas, BA: Bahia, CE: Ceará, MA: Maranhão, PE: Pernambuco, PB: Paraíba, PI: Piauí, RN: Rio Grande do Norte, and SE: Sergipe; Central-West − DF: Federal District, GO: Goiás, MS: Mato Grosso do Sul, and MT: Mato Grosso; Southeast − ES: Espírito Santo, MG: Minas Gerais, RJ: Rio de Janeiro, and SP: São Paulo; South − PR: Paraná, RS: Rio Grande do Sul, and SC: Santa Catarina.

**Figure 3:**
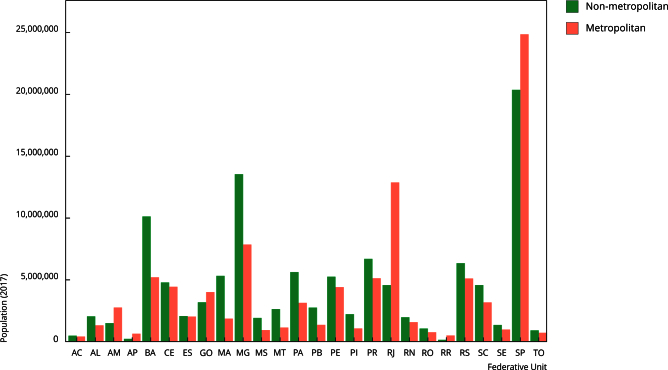
Population distribution by state: non-metropolitan vs. metropolitan area in Brazil.

Note: regions and their respective states: North − AC: Acre, AM: Amazonas, AP: Amapá, PA: Pará, RO: Rondônia, RR: Roraima, and TO: Tocantins; Northeast − AL: Alagoas, BA: Bahia, CE: Ceará, MA: Maranhão, PE: Pernambuco, PB: Paraíba, PI: Piauí, RN: Rio Grande do Norte, and SE: Sergipe; Central-West − DF: Federal District, GO: Goiás, MS: Mato Grosso do Sul, and MT: Mato Grosso; Southeast − ES: Espírito Santo, MG: Minas Gerais, RJ: Rio de Janeiro, and SP: São Paulo; South − PR: Paraná, RS: Rio Grande do Sul, and SC: Santa Catarina.

The decentralization index (*Ψ*) is shown in Figure 2. As expected, chikungunya was initially concentrated in metropolitan regions in most UF. However, six of the 27 UF deviated from this expected pattern: Amapá, where the incidence was 8.7 times higher in the non-metropolitan region than in the metropolitan region; Espírito Santo, 3.7 times higher; Paraná, 1.3 times higher; Sergipe, 2.7 times higher; Mato Grosso, 1.3 times higher; and Rio de Janeiro, 1.13 times higher. The population in these states is predominantly located in non-metropolitan regions (Figure 3). An exception is Rio de Janeiro state, where only 26.4% of the population lives in non-metropolitan region, although a large contingent of 4,231,306 individuals resides there.

To facilitate the interpretation of these findings, the data were stratified by region. Figure 2 shows the trends from this initial period until 2022. In the Northeast Region, the 2014 chikungunya epicenter was concentrated within metropolitan regions of all states except Sergipe State. Sergipe initially exhibited a higher incidence rate (*Ψ > 1*) in its non-metropolitan regions. However, beginning in 2016, Sergipe experienced a shift, with metropolitan municipalities exhibiting higher chikungunya incidence in most subsequent years. This pattern persisted across most states over time, punctuated by sporadic increases in non-metropolitan activity. This intermittent resurgence was observed in non-metropolitan regions of Paraíba, Alagoas, Rio Grande do Norte, Maranhão, and Ceará. Conversely, Bahia demonstrated a more sustained incidence of reported cases in its non-metropolitan region. Pernambuco and Piauí were the last to record a higher incidence in the non-metropolitan region, more recently in 2021 and 2022.

In the North Region, all states except one had a higher chikungunya incidence in their metropolitan regions at the beginning. The exception was Amapá, as described above, where the entry point was Oiapoque, on the border with French Guiana. Over time, the disease predominantly affected metropolitan regions. However, some years showed higher notification rates in non-metropolitan regions. An example is the state of Amazonas, where incidence increased progressively in non-metropolitan municipalities, reaching a peak in 2022. In contrast, Tocantins consistently reported a higher incidence of chikungunya in metropolitan regions, despite having a larger population residing in non-metropolitan areas (54.4%).

In the Central-West Region, the three states showed different behaviors. Mato Grosso had a higher incidence in the non-metropolitan region during the first two years. However, in 2017, there was an increase in cases in the metropolitan region. Goiás consistently reported a predominantly higher incidence in the non-metropolitan region over eight years, although an increase in metropolitan cases was observed in 2022. Mato Grosso do Sul had a different pattern, with cases predominantly reported in metropolitan regions until 2020.

In the Southeast Region, São Paulo presented disease incidence initially concentrated in the metropolitan region, which shifted to non-metropolitan regions in 2018, where it remained high until 2022. In Rio de Janeiro, the incidence was initially slightly higher in the non-metropolitan region. In 2017, the metropolitan region showed higher incidence rates, a trend that persisted until 2019, after which higher incidence returned to non-metropolitan regions, mirroring the trends in Minas Gerais and São Paulo. Espírito Santo, which has a population distribution approximately equal between non-metropolitan regions (50.5%) and metropolitan regions (49.5%), had the highest incidence in non-metropolitan regions until 2015. However, the metropolitan region showed higher rates in the following years. Similar to Bahia in the Northeast and neighboring Minas Gerais, it has shown a pattern of consistently higher incidence in non-metropolitan regions since 2015.

The South Region detected the lowest number of chikungunya cases nationwide (Table 1). Paraná consistently recorded a higher incidence in non-metropolitan municipalities, where 56.6% of its population also resides. The situation was reversed in 2019, when the metropolitan region showed higher incidence, but this returned to its previous pattern the following year. In contrast, Santa Catarina maintained higher incidence rates in its metropolitan regions throughout the study period. Rio Grande do Sul initially showed a slight predominance of cases in metropolitan regions but has recorded higher incidence in non-metropolitan municipalities since 2018.

## Discussion

Descriptive epidemiological studies of emerging infectious diseases form the basis for a better understanding of their dissemination dynamics ^1^ ^,^ ^2^ ^,^ ^3^. The dissemination of the CHIKV in Brazil revealed distinct introduction and transmission decentralization patterns among its UF. This study demonstrated that the disease initially had a higher incidence in metropolitan regions and progressively reached non-metropolitan regions at different times in each state, except for Tocantins and Santa Catarina. The results support the hypothesis that the decentralization index (*Ψ*) increased during the analyzed period in most locations, indicating a dispersion of transmission to non-metropolitan municipalities.

The pattern of decentralization of chikungunya throughout the study period can be explained by infrastructural, demographic, population mobility, and climate factors ^13^ ^,^ ^15^ ^,^ ^31^ ^,^ ^35^.

The control of urban arboviruses transmitted by *Aedes* remains a major challenge for public health. Even in large metropolitan centers, where resources, infrastructure, and technical capacity are comparatively greater, infestation and recurrent epidemics continue to occur ^12^ ^,^ ^36^, particularly in pockets of poverty characterized by irregular water supply and inadequate waste management ^3^ ^,^ ^27^ ^,^ ^28^. These structural vulnerabilities make such areas equally or even more susceptible to arboviral transmission than smaller or less densely populated municipalities. Cities such as Salvador (Bahia State) and Recife (Pernambuco State) recorded the presence of the virus in the initial forecasts of its introduction ^1^ ^,^ ^2^, possibly due to population agglomeration and greater connectivity of these regions with other states and countries. Smaller municipalities, in turn, face distinct challenges, such as limited financial and human resources to sustain continuous control actions, lower diagnostic capacity, and reduced risk perception among the population. Thus, despite their contextual differences, both large urban centers and smaller municipalities share structural barriers that hinder the implementation of effective and sustainable vector control measures.

Population mobility may have been a major factor in the initial spread of the disease. The hosting of a major international sporting event, the FIFA World Cup, in Brazil in 2014 also increased the circulation of travelers, thereby increasing the possibility of the virus entering these urban centers ^37^ ^,^ ^38^. These aspects are similar to the initial pattern of chikungunya that had already been observed in previous dengue epidemics, reinforcing the role of metropolises as the main “entry points” for new diseases. In addition, similar dynamics have been documented in other contexts, such as the diffusion of measles in São Paulo during the 2019 ^39^ epidemic and of COVID-19 in Rio de Janeiro ^40^, which followed a “center-periphery” diffusion pattern through urban connectivity networks. These parallels further underscore the importance of inter-city mobility and hierarchical connections in shaping the spatial dissemination of infectious diseases in Brazil.

Additionally, climatic factors, including high temperatures and irregular rainfall patterns, create ideal conditions for vector reproduction in both non-metropolitan and metropolitan regions. This decentralization pattern, specific to the Brazilian context, differs from that observed in other Latin American countries, where transmission remains predominantly concentrated in densely populated metropolitan regions ^36^ ^,^ ^41^.

As hypothesized in this study, most states reported a higher disease incidence in metropolitan regions during the initial emergence of the disease in Brazil. However, in some states, the introduction of the disease occurred in non-metropolitan regions, suggesting the existence of specific local risk factors. In Amapá, the presence of an international border played a significant role, as proximity to French Guiana, where the virus was already circulating ^1^ ^,^ ^42^, facilitated the introduction and spread of the virus through cross-border commerce and goods transportation. A similar process occurred in Sergipe, a small state internally bordered by the states of Alagoas and Bahia, where there was strong circulation of chikungunya. High connectivity with these neighbors resulted in a notable history of dengue virus circulation in the years preceding the introduction of CHIKV, attributed to the presence of the mosquito *Ae. aegypti* ^12^ ^,^ ^43^. In Mato Grosso, highway corridors connecting non-metropolitan regions to other states, as well as the previous circulation of *Ae. aegypti* in small population centers, facilitated the introduction of the virus outside the capitals ^44^. In Rio de Janeiro, commercial and industrial connectivity in regions such as the northern part of the state contributed to the emergence of initial cases far from the metropolis ^41^. In Espírito Santo, the relatively balanced population distribution between metropolitan and non-metropolitan regions, combined with worker displacement between rural and urban areas, facilitated the spread of chikungunya in smaller municipalities before the capital registered a higher incidence ^45^. These aspects highlight the importance of local characteristics and mobility routes in the dynamics of arbovirus introduction. Finally, the rapid decentralization can also be attributed to the tourist attraction of non-metropolitan regions ^46^, such as Paraná, due to the Iguaçu Falls (Foz do Iguaçu), which attract a large contingent of visitors from other regions where the virus is endemic, thus introducing the pathogen. Moreover, this region borders Paraguay and Argentina, which have reported high numbers of chikungunya cases ^47^.

Some limitations of this study relate to the quality and availability of the analyzed data. The completeness of epidemiological records varies across regions, states, and municipalities, and underreporting is usually more pronounced in smaller municipalities due to limited health infrastructure and restricted access to diagnostic services ^13^. These factors may lead to distortions in the distribution of reported cases between metropolitan and non-metropolitan areas, potentially influencing the calculation of the decentralization index.

Additionally, this study did not perform age- and sex-standardization of incidence rates, as the analyzed period corresponds to the initial expansion phase of an emerging disease, during which the population was broadly susceptible and the focus was on territorial and mobility-related factors.

Moreover, until the end of 2015 ^48^, specific diagnostic tests for chikungunya were not widely available in Brazil, which frequently resulted in cases being misclassified as dengue due to their clinical similarity ^28^. In addition, inconsistencies in case records and laboratory confirmations, with varying magnitude across regions, reinforce the possibility of differential bias. Finally, the scarcity of information regarding CHIKV virulence and population susceptibility ^32^ over time limits the understanding of outbreak recurrence and the evaluation of the effectiveness of public health strategies ^46^ ^,^ ^49^. Regarding the decentralization index proposed in this study, while it makes significant contributions to surveillance, it is important to note that its results may be influenced by fluctuations in municipalities with small populations or very low incidence. In the absence of sensitivity analyses or direct comparisons with traditional surveillance models, the findings should therefore be interpreted alongside complementary information, such as case series.

This study presents a comprehensive analysis of the spatial dynamics of chikungunya in Brazil, highlighting the decentralization index as an innovative tool to assess the regional spread of transmission. The use of national data over nearly a decade reinforces the robustness of the conclusions, highlighting the need for surveillance strategies tailored to various regional contexts and the strengthening of control actions for emerging infectious diseases.

## Conclusion

This study operationalizes an indicator for the surveillance of (re)emerging diseases in Brazil, the decentralization index (*Ψ*). Here, we apply it to chikungunya, combining epidemiological and demographic data and providing a synthesized view of disease activity at the national level. The use of this index revealed distinct dissemination patterns between metropolitan and non-metropolitan contexts, underscoring the importance of territories often neglected in arbovirus control and analyses. Beyond advancing the scientific understanding of the spatial dynamics of chikungunya, the decentralization index demonstrates practical applicability in the field of public health, as it can inform policy formulation and support decision-makers in resource allocation, strengthening surveillance actions, and prioritizing specific interventions in high-risk areas. Thus, the proposed approach reinforces the need for strategies adapted to regional inequalities, acknowledging both historically central regions and those less visible in monitoring and control efforts.

## Data Availability

The databases used in the study, including extraction codes, analyses, and results, are available in the repository: http://github.com/iasmimalmeida/Decentralization-index.
