# Supplementary material for: Beyond the metropolises: the decentralization of chikungunya to non-metropolitan areas across Brazil
Source: Cad Saude Publica. 2026 Jul 6;42:e00129025. doi: 10.1590/0102-311XEN129025 (PMC13335780; doi:10.1590/0102-311XEN129025)
Supplement: Supplementary Material [file 1678-4464-csp-42-EN129025-s.pdf]

Supplementary material

Figure S1 Spatial distribution and temporal evolution of chikungunya incidence in Brazil (2014-2022).

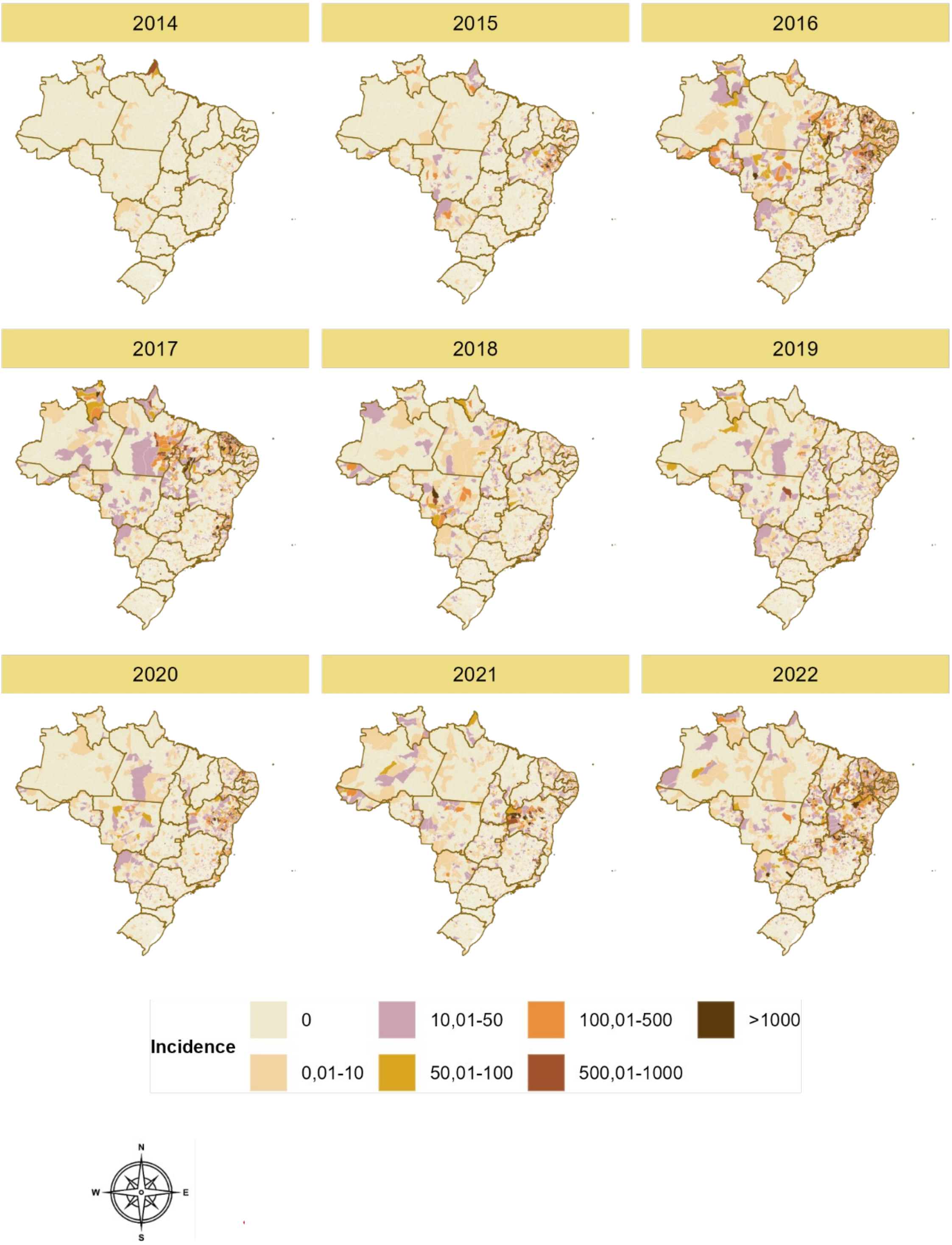

Note: Annual maps showing the incidence (cases per 100,000 inhabitants) of Chikungunya in Brazilian municipalities, highlighting the increase and spread of the disease over this period.
